# Lipid metabolism: the potential targets for toxoplasmosis treatment

**DOI:** 10.1186/s13071-024-06213-9

**Published:** 2024-03-06

**Authors:** Tian-Yi He, Ye-Tian Li, Zhen-Di Liu, Hao Cheng, Yi-Feng Bao, Ji-Li Zhang

**Affiliations:** Health Science Center, Ningbo University, Ningbo, China

**Keywords:** *Toxoplasma gondii*, Toxoplasmosis, Lipid metabolism, Fatty acids, Phospholipids, Cholesterol

## Abstract

Toxoplasmosis is a zoonosis caused by *Toxoplasma gondii* (*T. gondii*). The current treatment for toxoplasmosis remains constrained due to the absence of pharmaceutical interventions. Thus, the pursuit of more efficient targets is of great importance. Lipid metabolism in *T. gondii*, including fatty acid metabolism, phospholipid metabolism, and neutral lipid metabolism, assumes a crucial function in *T. gondii* because those pathways are largely involved in the formation of the membranous structure and cellular processes such as division, invasion, egress, replication, and apoptosis. The inhibitors of *T. gondii*’s lipid metabolism can directly lead to the disturbance of various lipid component levels and serious destruction of membrane structure, ultimately leading to the death of the parasites. In this review, the specific lipid metabolism pathways, correlative enzymes, and inhibitors of lipid metabolism of *T. gondii* are elaborated in detail to generate novel ideas for the development of anti-*T. gondii* drugs that target the parasites’ lipid metabolism.

**Graphical abstract:**

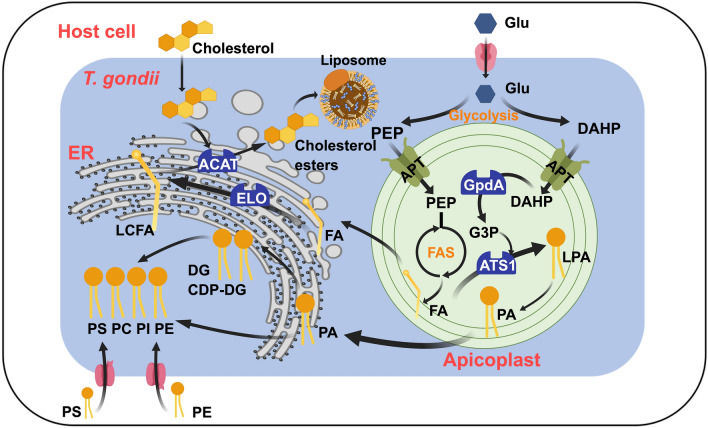

## Background

Toxoplasmosis is a zoonosis attributed to *Toxoplasma gondii* (*T. gondii*), which can infect both animals and humans [1]. *T. gondii* infection is commonly caused by consuming contaminated food [2]. Acute *T. gondii* infection typically presents as asymptomatic in individuals with a competent immune system. However, toxoplasmosis can lead to significant ramifications in individuals with compromised immune systems [3]. Pregnant women who are infected with toxoplasmosis may vertically transmit the parasites to their fetus, causing miscarriages, malformations, etc. [4]. Toxoplasmosis is considered to be a highly influential and devastating parasitic disease due to its wide prevalence of ~ 2 billion individuals worldwide [5].

Regrettably, the therapeutic interventions for toxoplasmosis are still limited, due to the complicated life cycle of *T. gondii*. Sulfadoxine-pyrimethamine, the gold standard of clinical treatment for toxoplasmosis, has significant adverse effects in patients [5]. Thus, the search for alternative pharmacological agents presenting reduced toxicity and enhanced safety profiles remains an ongoing and challenging endeavor.

As an intracellular parasite, *T. gondii* relies on membranous structures such as parasitophorous vacuole membrane (PVM) for invasion, proliferation, and egress [6, 7]. Invasion occurs with the formation of the PVM, and after many rounds of replication, the parasites rupture the parasitophorous vacuole (PV) and egress into the extracellular environment [6]. In addition, lipids act as the important components of cell membranes or organelle membranes, as well as the signaling molecules in intracellular messaging [8]. Therefore, the enzymes involved in lipid metabolism pathways in *T. gondii* specifically would be ideal targets to treat toxoplasmosis without affecting the host [9]. In this review, the specific pathways, related enzymes, and inhibitors involved in fatty acid metabolism, phospholipid metabolism, and neutral lipid metabolism are reviewed with the aim of providing new insights for the development of lipid metabolism-targeting drugs (Table 1).

## Fatty acid metabolism

**Fig. 1:**
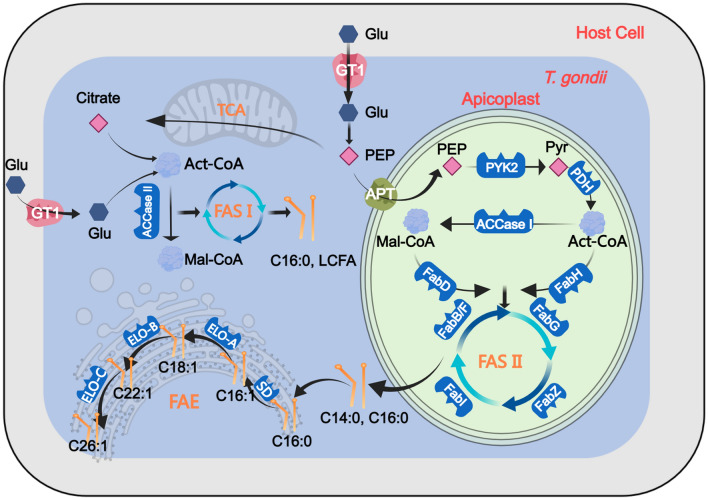
Fatty acid metabolism progress in *T. gondii*

By far, researches have shown that FA synthesis de novo derives from three pathways (Fig. 1): (A) type II fatty acid synthesis (FAS II) generating short-chain fatty acids (C14:0 and C16:0) in apicoplast, (B) FA elongation (FAE) generating long-chain fatty acids (LCFA) (C16:1, C18:1, C22:1, and C26:1) in endoplasmic reticulum (ER), and (C) type I fatty acid synthesis (FAS I) generating C16:0 or LCFA in cytoplasm [10].

In the beginning of the FAS II pathway, phosphoenolpyruvate (PEP) is generated in the cytoplasm by the glycolysis pathway and then transported via the apicoplast phosphate transporter (APT) [11–13]. Inside the apicoplast, PEP can be converted by pyruvate kinase 2 (PYK2) to pyruvate (Pyr), which in turn serves as a substrate for apicoplast pyruvate dehydrogenase (PDH) to produce acetyl-CoA (Act-CoA) for fatty acid synthesis [14, 15]. Then, Act-CoA is carboxylated to malonyl-CoA (Mal-CoA), which is the other essential substrate for the FAS II pathways, under the consumption of bicarbonate and ATP by acetyl-CoA carboxylase I (ACCase I) [12, 16]. The fatty acyl of Mal-CoA and Act-CoA is bonded to ACP and participates in elongation. Finally, these fatty acids generated by the FAS II pathways will be transferred to the cytosol [16]. Apart from FAS II pathway, the FAE pathway consists of three fatty acid elongases, two reductases, and a dehydratase in the ER. FAS II-synthesized C16:1 is converted to C18:1 via the activities of fatty acid elongase A (ELO-A), while fatty acid elongase B (ELO-B) elongates ELO-A-generated C18:1 to C22:1, and fatty acid elongase C (ELO-C) elongates ELO-B-generated C22:1 to C26:1 in two carbon increments [11, 17]. In addition, FAS I is involved in the synthesis of C16:0 and the elongation of LCFA.

### FAS I pathway

*T. gondii*’s FAS I pathway, containing one fatty acid synthase (FAS) and two polyketide synthases (PKSs) (Table 1), involves the synthesis of palmitic acid (C16:0) and the elongation of LCFA. In addition, distinct from mammals, which only have the FAS I pathway generating all the FAs, *T. gondii* has three pathways. Some analyses of the FAS I pathway in apicomplexans have also revealed structural differences compared with those of mammals [13]. Mammalian FAS is a unimodular protein composed of seven enzymatic domains. These enzymatic domains act sequentially for several cycles of elongation on an acyl moiety attached to ACP [13]. By contrast, FAS in *T. gondii* is a multimodular protein where each module contains a set of multiple enzymatic domains [17, 18]. It is likely that the substrate is undergoing a single round of elongation in each module prior to being transferred to the ACP of the next module [13].

Although the studies focusing on this pathway are limited, FAS I is considered a potential target, given its unique role in the synthesis of oocysts and the significant difference between *T. gondii* and mammals.

### FAS II pathway

The FAS II pathway (Fig. 1) obtains enzymes that synthesize myristic acid (C14:0) and palmitic acid (C16:0) de novo [9]. In apicomplexans, C14:0 and C16:0 are found in phosphatidyl choline (PC) and are used as building blocks by the FAE to make LCFA [19]. The myristylation and palmitoylation by FAS II also contribute to the gliding machinery of *T. gondii* when invading. Importantly, humans rely on FAS I for the synthesis of LCFA instead of FAS II [11], so blocking the FAS II pathway in *T. gondii* does not hinder fatty acid synthesis in humans. Thus, the key enzymes in FAS II pathway can be potential targets combating toxoplasmosis.

ACP (Table 1), which is a core protein of the FAS II pathway [18], is required for the final formation of C14:0 and C16:0. In the process of elongation, ACP is bonded to the growing fatty acyl radicals and participates in fatty acyl elongation, which consists of cyclic sequential reactions catalyzed by a series of enzymes [13]. The absence of this core substance would terminate FAS II, leading to apicoplast segregation deficiencies and proliferation defects [20]. Therefore, the components specifically targeting ACPs have an anti-proliferative effect.

FabI is responsible for the final reductive step in the FAS II pathway, and there is no homolog to FabI in mammals [21]. Triclosan is a potent inhibitor of *Tg*FabI (Table 1) [22], the inhibitory activity of which is binding to a conserved Tyr residue in the active site and preventing the formation of a ternary complex [23]. After binding to inhibitors, the catalytic activity decreases, leading to apicoplast morphology alterations as well as division defects [19]. Moreover, the modifications of the A-rings and B-rings of triclosan can improve the absorption, distribution, metabolism, excretion, and toxicity (ADMET) profiles, increasing the drug’s premeability [23].

PDH (Table 1) is another key enzyme involved in the formation of Act-CoA [12]. A heterotetrameric E1α domain in the PDH complex catalyzes the decarboxylation of pyruvate to initiate the whole reaction [18]. However, despite slower growth, mutants lacking the E1α and E2 subunits of *Tg*PDH were viable in vitro and in vivo, demonstrating the dispensability of PDH for parasite survival and virulence [18].

### FAE pathway

Fatty acids produced by FAS II in *T. gondii* are released into the cytosol and elongated in the ER [11, 24]. Compared with short-chain FAs, long-chain and unsaturated FAs are able to fit in different membrane fluidity [25]. In addition, long-chain FAs can be converted into the phosphatidic acid intermediate lysophosphatidic acid (LPA) by glycerol 3-phosphate acyltransferase (*Tg*ATS1) (Table 1) [9]. Apart from participating in the formation of phospholipids, unsaturated FAs can be oxidized to form eicosanoids [26]. To sum up, the FAE pathway (Fig. 1) appears to have irreplaceable functions in generating C16:1, C18:1, C22:1, and C26:1 [10, 18].

Three fatty acid elongases (ELOs) are crucial in the FA elongation pathway, among which ELO-A converts C16:1 to C18:1, after which ELO-B elongates C18:1 to C22:1, and finally ELO-C elongates C22:1 to C26:1 (Table 1) [11]. This suggests that all the ELOs have substrate specificity [6]. Indeed, evidence for ELOs as antiparasitic targets was provided by the growth inhibition and weakened virulence of *T. gondii* upon interference with two or all the ELOs.

During the synthesis of LCFA, ELOs are linked to hydroxyacyl-CoA dehydratase (DEH) or enoyl-CoA reductase (ECR). Removal of DEH and ECR could result in the inactivation of all ELO complexes, leading to a marked reduction in the C22:1 and C26:1 FAs [17]. Additionally, lacking LCFA coincides with an apparent decrease in phosphatidyl inositol (PI) and phosphatidyl ethanolamine (PE) [17].

In addition to the role FAE pathway playing in the synthesis of LCFA, the sequence of genes encoding ELOs in *T. gondii* and humans are quite distinctive. Therefore, the components involved in FAE are also considered promising therapeutic targets. However, the structures and active sites of these enzymes are not fully understood, and further investigations are demanded.

## Phospholipid metabolism

There is a tight junction between fatty acid synthesis and phospholipid synthesis. *Tg*ATS1 controls the process that some FA chains bind glycerol 3-phosphate (G3P) to generate LPA [9], which is precursor of bulk phospholipids.

Phospholipids can be mainly divided into glycerol phospholipids and sphingolipids, which act as constituents of cell membranes as well as the signaling molecules [8]. Interestingly, *T. gondii* synthesizes phospholipids de novo in a different way from mammals, which contributes to parasite viability at various life stages [13]. The investigation of hypothetical enzymes in phospholipid metabolism provide opportunities to explore potential drug targets combating *T. gondii*.

### Glycerol phospholipid metabolism

**Fig. 2:**
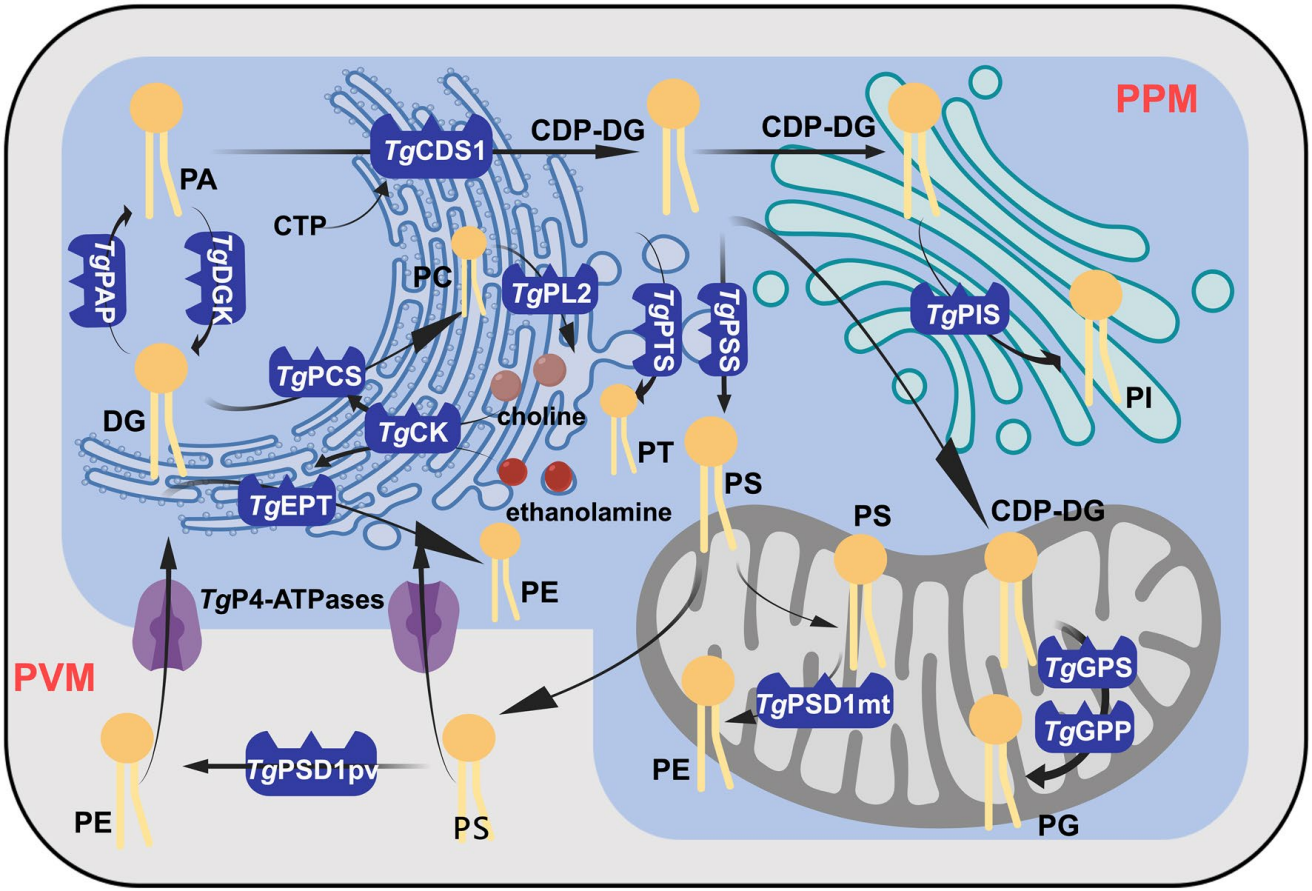
Glycerophospholipid synthesis pathway in *T. gondii*

As cell membrane components, glycerol phospholipids participate in nearly all cellular processes [27]. Though different kinds of glycerol phospholipids seem to be independent of each other, there are intersections and compensations between their synthetic paths (Fig. 2). Phosphatidic acid (PA) and diacylglycerol (DG) can be interconverted and maintained in balance [28], with the former forming cytosine diphosphate diacylglycerol (CDP-DG) pools catalyzed by cytosine diphosphate diacylglycerol synthetase (*Tg*CDS) and the latter synthesizing PC and PE (Table 1) [29–31]. CDP-DG can be transported to the Golgi for the PI synthesis, or to the mitochondria for the PG synthesis [29], as well as in the ER for the synthesis of PT and PS [32, 33]. Afterward, PS can be transported to either vacuoles or mitochondria, where it undergoes decarboxylation to form PE [34]. Meanwhile, *T. gondii* can take up PS and PE from the external environment via P4-ATPases on the plasma membrane [35].

DG and PA are the most initial substrates for phospholipid metabolism. DG links extracellular and intracellular events, leading to important biological processes [36]. Diacylglycerol kinases (DGKs) regulate the intracellular levels of two second messengers by converting DG to PA (Table 1) [28]. PA participates in gliding motility and invasion invasion as well as releasing adhesin complexes and proteases that are implicated in programmed egress [36].

The delicate balance between DG and PA is controlled by *Tg*LIPIN (Table 1), which regulates the flux between lipid storage and membrane biogenesis as well as release of micronemes [37]. DGK-1 depletion or inhibition of the enzyme by R59022 impairs egress and micronemes secretion (Table 1), further causing parasite death [38]. Meanwhile, propranolol promotes microneme secretion by inhibiting phosphatidic acid phosphatase (*Tg*PAP) to decrease DG synthesis and increase PA content (Table 1) [38]. Research also revealed that the calcium-dependent protein kinase (*Tg*CDPK7) activates the pathway for PA synthesis from FAs by regulating glycerol-3-phospahte-acyl transferase (*Tg*GPAT) and possibly *Tg*PAP (Table 1) [39].

Large amounts of DG are used to synthesize PE and PC. The former glycerophospholipid species, PE, is synthesized primarily by the CDP-ethanolamine pathway. It is successively catalyzed by *Tg*EK, *Tg*ECT, and *Tg*EPT (Table 1). Among them, *Tg*ECT is localized in the cytoplasm, and its conditional knockdown leads to a reduction in PS, PE, and PT [31]. Apart from CDP-ethanolamine pathway, PE can also be obtained by the decarboxylation of PS, which is catalyzed by two phosphatidyl serine decarboxylases (*Tg*PSDs) in PV and mitochondria, respectively (Table 1) [34]. Among them, *Tg*PSD1pv converts PS into PE outside the plasma membrane, which reduces the chances of macrophages killing *T. gondii* by recognizing PS, as well as expanding the space for tachyzoite replication by accelerating PVM synthesis by utilizing PE [40], whereas another enzyme, *Tg*PSD1mt, converts PS to PE in the mitochondria [40].

Another DG-derived glycerol phospholipid synthesis pathway, CDP-choline pathway, is initiated by choline kinase (*Tg*CK) (Table 1), which phosphorylates both choline and ethanolamine. Unexpectedly, upon inhibition of *Tg*CK, the mutant strain was not impaired in its growth and even exhibited a normal PC biogenesis [30], reflecting the flexibility and plasticity of PC synthesis in *T. gondii*. Dimethylethanolamine (Etn(Me)(2)), a choline analog, significantly interferes with the PC metabolism of *T. gondii*, resulting in abnormal membrane structure formation and markedly inhibiting its growth within host cells [41].

Under the catalysis of *Tg*CDS1, PA involves the synthesis of CDP-DG pools for further production of phospholipids such as PS, PT, PI, and PG [28]. Unlike in humans, *T. gondii* can obtain serine as well as threonine from the host cells and synthesize them into PS and PT. The distribution of PS across the plasma membrane is important for microneme exocytosis and immune escape from host [33]. *T. gondii* may have evolved PT as a specialized variant of PS, loss of which depletes calcium stores in tachyzoites and dysregulates calcium release [32]. Unexpectedly, the parasite replication was normal after the genetic disruption of *Tg*PTS (Table 1); however, the egress and invasion processes were inhibited and its virulence was reduced [42], suggesting PS may involve in the calcium flux across the plasma membrane and calcium storage organelles [43].

Two distinct CDP-DG pools also need to be transported to the Golgi apparatus and mitochondria to synthesize PI and PG, respectively [27, 29]. This spatial distribution of lipid synthesis enables coordinated lipid transport by organelles within *T. gondii*. Upon inhibition of CDP-DG synthetase *Tg*CDS, the amounts of PI and PG are dramatically reduced, leading to organelle dysfunction and growth inhibition [32]. Of interest, a set of active serine hydrolases (*Tg*ASH) identified within *T. gondii* are thought to play an important role in the conversion of PA to CDP-DG. Upon inhibition of these enzymes by JCP341, JCP342, JCP343, JCP348, and JCP383, PA was significantly elevated and CDP-DG was decreased in *T. gondii*, thereby affecting downstream phospholipid metabolism (Table 1) [44]. *T. gondii* can import and utilize both inositol and endogenous CDP-DG to synthesize PI [27]. PI can be made into PI3-monophosphate and PI3,5-diphosphate, which are required for apicoplast biogenesis. The knockdown of the PI synthetase (*Tg*PIS) leads to an increase in PS and PG levels, facilitating the lytic cycle breakdown (Table 1) [27]. The role of PG in *T. gondii* is unclear, but it usually serves as an intermediate for mitochondrial endosomal membrane biogenesis and cardiolipin synthesis [29].

The process of glycerophospholipids scavenging from the host is also of considerable importance. *T. gondii* acquires precursor material from the host for the synthesis of major phospholipids, mainly PC, PE, PS, and PI, as well as several unknown lipids, suggesting that recycling of the building blocks scavenging from the host coexists with de novo pathway [45]. The membranes of the PV are tightly bound to the host cell’s lipid biosynthetic apparatus (i.e., the ER and the mitochondrion), which promotes access to lipids [45]. The absence of PE and PS in *T. gondii* can be compensated by the transportation from the host cells via P4-ATPase [35], thereby better adapting to environmental changes. Recent studies showed that *Tg*P4-ATPase1 and *Tg*Lem1 work in coordination to translocate PS during the lytic cycle of *T. gondii*. The conditional knockout of P4-ATPase1 and Lem1 severely disrupt the asexual reproduction, demonstrating the vitality of lipid flipping translocases (Table 1) [46]. PI species with shorter chains (C30/C32/C34) are synthesized de novo, while those with longer acyl chain (C38/C40) are salvaged from host cell, which seems to generate adequate PI for IP_3_-dependent Ca^2+^ signaling in the parasite [27].

The unique glycerophospholipid degradation system in *T. gondii* prevents it from death due to lipid accumulation. For instance, PC phospholipase plays a crucial role. D609, an inhibitor of PC-specific phospholipase C, restrains the proliferation and growth of *T. gondii* (Table 1) [47]. Additionally, the absence of *Tg*PL2, a patatin-like PC phospholipase, resulted in an abnormal build-up of membranous structures, indicating that lipid homeostasis was compromised [48]. It is also found that treatment of *T. gondii* with atglistatin led to a decrease in parasite replication proportional to drug concentration and disorganization of the parasites by acting on one or more of the six parasite-derived patatin-like phospholipases (Table 1) [26].

The sensitivity of enzymes in *T. gondii*’s glycerophospholipid synthesis shows a new way to disrupt the parasite’s lytic cycle. Its significance also makes them possible drug targets for toxoplasmosis treatment.

### Sphingomyelin metabolism

Sphingomyelin acts as a component in cell membranes and signaling messengers, participating in replication and apoptosis as well as the formation of lipid rafts [26]. Remarkably, de novo synthesis of sphingolipids in *T. gondii* is rather critical, as deprivation of sphingomyelin uptake has no significant effect on *T. gondii* [49].

Serine palmitoyl transferase (SPT) is the first rate-limiting enzyme in sphingomyelin biosynthesis [50]. L-cycloserine, an inhibitor of *Tg*SPT, can strongly inhibit *T. gondii* growth (Table 1) [51]. Although the interference of de novo sphingomyelin synthesis has little effect on acute infection, it dramatically alleviates the cyst burden in the brain of chronically infected mice [52]. Ceramide, which is modified into sphingomyelin in mammalian cells, is used for the synthesis of inositol phosphorylceramide (IPC) in fungi and plants. Interestingly, both sphingomyelin and IPC are found in *T. gondii* [53]. IPC in *T. gondii* is catalyzed by *Tg*SLS (Table 1), which is a functional homolog of yeast IPC synthase (AUR1p) [49]. AbA has now been discovered to not target this pathway [54]. Nevertheless, given the status of IPC synthase as a promising drug target among the fungi, the identification of *Tg*SLS implies a unique sphingolipid synthesis step existing in *T. gondii* and furthermore offers the possibility of targeting this enzyme with novel antiprotozoal agents.

Apart from de novo synthesis, *T. gondii* may also scavenge sphingomyelin or their precursors from the host cell [49]. It has been reported that the ceramide phosphorylethanolamine (CPE) and sphingomyelin found in intracellular tachyzoite may due to the aggregation of non-abundant lipids derived from the host [55]. Notably, while *T. gondii* ingests lipids, it affects host sphingolipid metabolism in turn. Among them, host sphingosine kinase expression is stimulated, whereas three genes encoding sphingomyelin synthases are significantly inhibited [55].

Understanding the sphingomyelin and IPC synthesis pathway in *T. gondii* is of particular importance, yet research in this area remains limited. Hence, the investigation into sphingomyelin synthase and *Tg*SLS holds considerable potential for future research endeavors.

## Neutral lipid metabolism

Neutral lipid metabolism is another metabolic process in *T. gondii*, which is related to its energy storage and host–pathogen interaction [56, 57]. Herein, we mainly describe the metabolism of cholesterol and triglyceride.

### Cholesterol metabolism

**Fig. 3:**
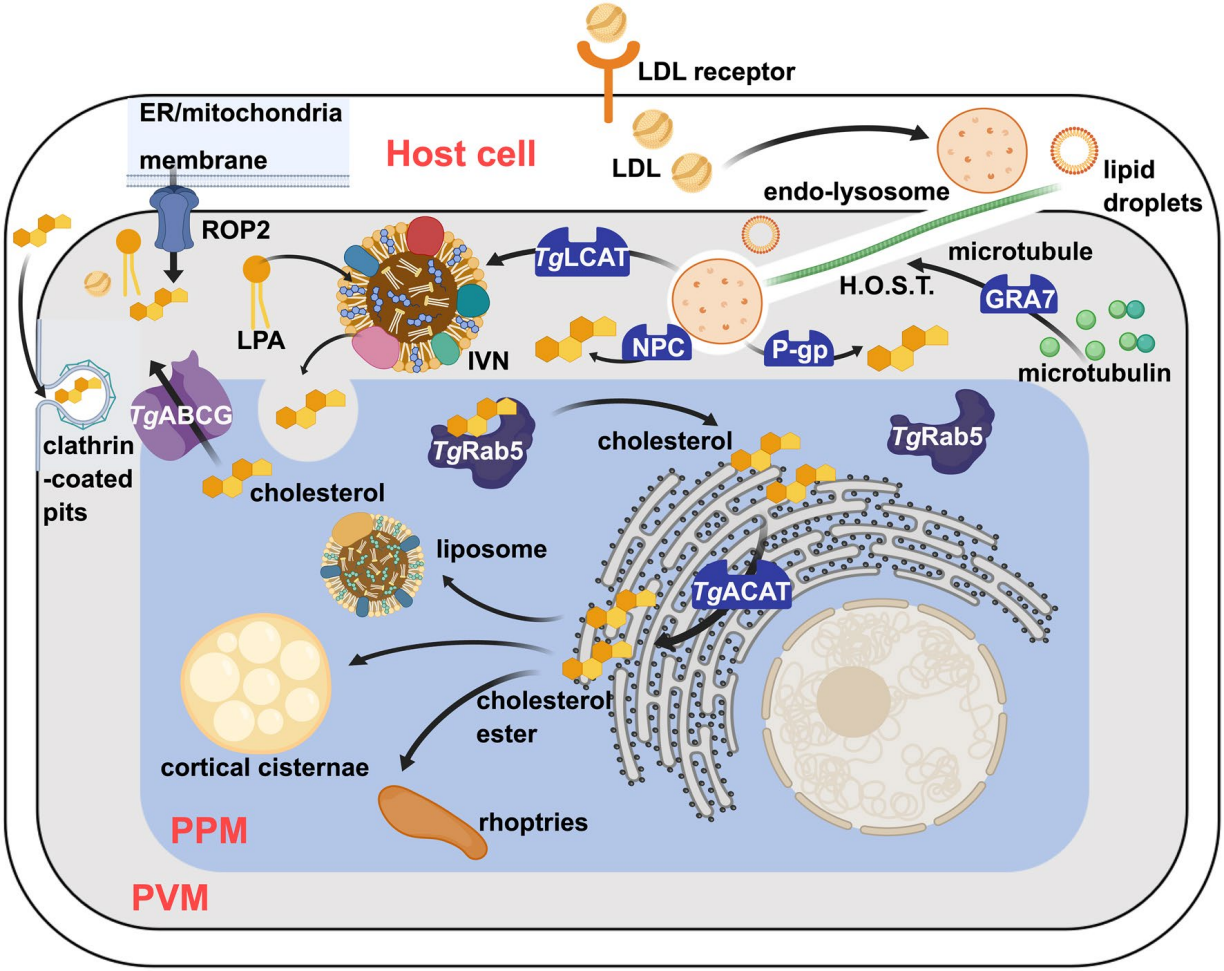
Overview of cholesterol scavenging and metabolism pathway in *T. gondii*

*Toxoplasma gondii* lacks enzymes for cholesterol biosynthesis de novo [56]. To satisfy its nutritional needs, *T. gondii* has evolved unique strategies to salvage this essential lipid from the host environment and has used them as raw materials for its own membrane biogenesis (Fig. 3). *T. gondii* scavenges endolysosomes and lipid droplets containing cholesterol from host cells through the host organelle-sequestering tubulostructures (HOST) structure, whose core microtubule component is synthesized by GRA7 [57]. These lipid-filled vesicles can be put into intravacuolar network (IVN) by lecithin: cholesterol acyltransferase (*Tg*LCAT) [58, 59], and their lysophospholipids can be broken down to release cholesterol. Cholesterol inside the PV may enter the PPM by endocytosis, while excess cholesterol may be exported to the PV through ATP-binding cassette G subfamily (*Tg*ABCG) [60]. The internalized cholesterol is transported through *Tg*Rab5 to the ER [61], where it transforms into cholesterol esters via the process of acyl-CoAs: cholesterol acyltransferase (*Tg*ACAT) (Table 1) [62]. Finally, they are distributed as cholesterol esters in different organelles, including liposomes, cortical cisternae, and rhoptries [57]. The impairment of cholesterol salvage is anticipated to have detrimental consequences on the proliferation of parasites, which can be exploited as a promising therapeutic approach for toxoplasmosis [56].

Among the various scavenging pathways, the low-density lipoprotein (LDL) endocytosis pathway has been intensely investigated. *T. gondii* gains access to the lipid content of cholesterol-filled host organelles by actively recruiting them around the PV [57]. HOST play an irreplaceable role in the process of endocytosis, which serves as conduits for the delivery of host endolysosomes and lipid droplets within the PV [57]. The formation and stabilization of HOST require the tubulogenic dense granule protein GRA7, which helps to form the dense electron-dense coat distributed on the HOST surface. Further research also observed impairments of growth in the GRA7 knockout parasites.

Following recruitment of liposomes through the HOST structure, the release of cholesterol from lipid droplets requires the host Niemann-Pick type C (NPC) protein (Table 1) [63], which mediates cholesterol egress across the endolysosomal membranes. Mutations in either the NPC1 or NPC2 proteins will lead to a massive accumulation of cholesterol in the core of lysosomes [63], suggesting that normal lysosomal motility is implicated in the cholesterol accessibility for the intracellular parasites. The close association between PV and host membranous organelles such as mitochondria and ER affords another mode of lipid translocation, probably through membrane translocators or channels that remain to be identified [64]. The maintenance of this direct contact relies on the parasite rhoptry protein ROP2 being anchored to the PV membrane [65].

The host lipid-rich organelles accumulated in PV frequently become enveloped by the tubules of the IVN, which consists of lipids and proteins initially secreted by the parasite and dramatically expanded with the salvage of host lipids [58]. The process of forming these structures is proposed to be associated with *Tg*LCAT [59], a dense granule protein secreted by *T. gondii* onto the IVN. The *Tg*LCAT phospholipase A2 (PLA2) activity product, namely lysophospholipids, may disrupt the lipid monolayer and thus liberate host lipids present in the host-derived vesicles for the parasites [59]. In addition to processing phospholipid, *Tg*LCAT could transfer the acyl groups from phospholipids to the 3-OH of cholesterol inside the PV, forming cholesteryl esters (CE) and thereby removing the excess cholesterol scavenged. LCAT-deficient *T. gondii* exhibited growth defects both in vitro and in mice, while parasites overexpressing *Tg*LCAT were more virulent than wild type parasites in mice [59]. More and deeper research needs to be put into *Tg*LCAT in search of target inhibitors. A P-glycoprotein (P-gp) seems to benefit cholesterol trafficking by promoting the egress of free cholesterol across the endolysosomal membranes (Table 1), as massive cholesterol accumulation was observed in P-gp-deficient cells [66].

Cholesterol incorporation into the parasite is largely abolished after treatment with various proteases, which sheds some light on the presence of a cholesterol transport system at the parasite plasma membrane (PPM) involving protein-binding sites [67]. Some ATP-binding cassette (ABC) G subfamily transporters have been identified as lipid translocators, among which *Tg*ABCG107 may be responsible for the cholesterol influx across the PPM [60]. However, the mechanisms through which *T. gondii* internalizes and digests material are not completely elucidated. Further investigation of these novel structures, including the identification of new molecular components involved, may provide new clues for anti-toxoplasmosis drug development. Other cholesterol originating from the host cell would be redistributed to various parasite cell compartments, including the plasma membrane, cortical cisternae, and rhoptries, as well as lipid droplets in the form of cholesterol esters. This dynamic movement between parasite organelles is mediated by *Tg*Rab5 [61].

To circumvent the toxic accumulation of free cholesterol in the *T. gondii* [56], *T. gondii* activates two enzymes (*Tg*ACAT1 and *Tg*ACAT2) that are dedicated to the synthesis of CE for storage in lipid bodies [62]. Blockade of cholesterol esterification with the ACAT inhibitors DuP 128 and CI 976 could result in the rupture of the plasma membrane and the death of *T. gondii* [62]. In addition, *T. gondii* has higher vulnerability toward ACAT inhibitors compared with mammalian cells [62]. The esterification and storage of cholesterol catalyzed by two ACAT enzymes may free *T. gondii* from constant cholesterol uptake and help it better survive in a host environment where cholesterol concentrations vary intermittently.

Meanwhile, degradation is another way to regulate lipid homeostasis and growth of *T. gondii*, though there is no evidence for enzymes that catalyze lipolysis of cholesterol esters [43]. The previously mentioned active serine hydrolases (*Tg*ASH) is a potential cholesterol lipolytic enzyme, as *T. gondii* exhibits excessive cholesterol and oleic acid when treated with small molecule inhibitors or in the absence of *Tg*ASH4 [43].

### Triglyceride metabolism

*Toxoplasma gondii* contains triglyceride in addition to cholesterol esters [68]. Stored triglyceride may be a reservoir of fatty acids utilizable for phospholipid biosynthesis and exploitable as respiratory substrates [69]. Significantly, the triglyceride metabolism of *T. gondii* exhibits a close association with that observed in humans. When *T. gondii* proliferates, the serum triglyceride content in the human body will be significantly reduced, threatening patient’s health [70], hinting that triglyceride metabolism in *T. gondii* exhibits an instrumental role in parasite–host interactions. Therefore, there has been an increasing focus on investigating the triglyceride metabolism in *T. gondii*.

Triglyceride is mainly formed on the basis of diglycerol and oleic acid. Diacylgycerolacyl transferase (DGAT) plays a crucial role in limiting the synthesis of triglycerides. It is founded that disruption of the gene resulted in a reduction in triglycerides and the generation of nonviable parasites [71]. Unlike mammals, which have two DGAT enzymes, *T. gondii* has only one that plays a role in the ER, called DGAT1. Due to this difference, though the *Tg*DGAT1 enzyme is blocked, the host cell can still rely on another enzyme to make triglyceride [69]. Currently, *Tg*DGAT1 is a promising and hot target for drug development against *T. gondii* [72]. T863 is an inhibitor that has been found to have significant effects (Table 1). T863-treated parasites do not form LDL but instead build up large membranous structures within the cytoplasm [72]. The improper channeling and management of lipid excess leads to the ultrastructure of the ER, which displays a swollen appearance and occupies a large volume of the cytoplasm. The observations serve to emphasize the significant disruption in lipid homeostatic pathways and changes in parasite membrane organization brought about by T863, suggesting that DGAT is a promising target that can cause the death of *T. gondii* without affecting the host cells.

The investigation into the triglycerides of *T. gondii* is garnering a growing fascination. With increased confidence, the identification of the present inhibitors will serve as a catalyst for scientists to persist in their pursuit of more potent inhibitors while concurrently mitigating the deleterious side effects associated with the initial inhibitors.

**Table 1:** The promising targets and its inhibitors of lipid metabolism in *T. gondii*

Pathway	Target	Inhibitor
Fatty acid		
FAS I	FAS	
	PKS	
FAS II	ACP	
	PDH	
	FabI	Triclosan
FAE	ELO-A	
	ELO-B	
	ELO-C	
	ATS	
Glycerol phospholipids		
CDP-DG pool	CDS	
	DGKs	R59022
	LIPIN	
	PAP	Propranolol
	CDPK7	
	PTS	
	PIS	
CDP-ethanolamine	EK	
	ECT	
	EPT	
	PSD	
CDP-choline	CK	
	ASH	JCP341, JCP342, JCP343, JCP348, and JCP383
Scavenge	P4-ATPase	
	Lem1	
Degradation system	PC phospholipase	D609
	PL2	Atglistatin
Sphingomyelin	SPT	L-cycloserine
	SLS	
Cholesterol	GRA7	
	LCAT	
	ABCG	
	Rab5	
	ACAT	DuP 128 and CI 976
	NPC	
	P-gp	
Triglyceride	DGAT	T863

## Conclusions

**Fig. 4:**
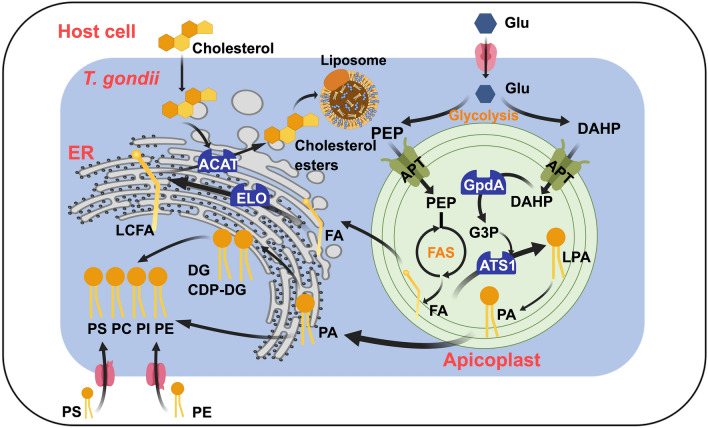
The relationship between fatty acid, phospholipid and cholesterol metabolism

The lipid metabolism in *T. gondii* can be categorized into three distinct parts (Fig. 4): (1) FAs can be synthesized de novo through FAS pathway mainly in apicoplast after glycolysis [9]; (2) *Tg*ATS1 adds G3P to a portion of FAs for the synthesis of intermediate LPA, which further synthesize DG and glycerophospholipid derivatives such as PC, PE, etc. [9]; and (3) some other FAs are catalyzed by ELO in the ER to generate LCFA, which, in conjunction with cholesterol, are utilized by ACAT enzyme to produce CE [73]. Enzymes such as *Tg*ATS1, DGK-1, and ACAT govern the flux of diverse lipid components and the delicate equilibrium between membrane formation and lipid storage [9, 17, 68]. The enzymes under consideration reveal species specificity, implying their distinctiveness from homologous enzymes found in mammalian cells.

Several enzymes related to lipid metabolism in *T. gondii* can be analogized to enzymes found in other parasites. Acetyl-CoA carboxylase, FabH, and FabI are all involved in the fatty acid metabolism of *T. gondii* and *Plasmodium falciparum* (*P. falciparum*) in the FAS pathway [74], illustrating the effectiveness in antiprotozoal drug development. The relative glycerophospholipids content in *T. gondii*, *P. falciparum*, *Trypanosoma brucei* (*T. brucei*), *Trypanosoma cruzi* (*T. cruzi*), and *Leishmania donovani* (*L. donovani*) are quite similar [55, 75–78]. PTS can be only found in *T. gondii*, *Eimeria falciformis* (*E. falciformis*), and *Neospora caninum* (*N. caninum*), revealing PT and its enzyme PTS is evolved to satisfy special taste for coccidian parasites [79]. *T. gondii* possesses ethanolamine phosphorylceramide (EPC), SM, and IPC, while *T. brucei* presents IPC and SM in the procyclic stage, as well as SM and EPC in the bloodstream stage [80], suggesting its stage-specific utilization of sphingophospholipid. As for neutral lipid metabolism, *Tg*DGAT1 is a potent target of *T. gondii* [69]. Interestingly, the amounts of triglycerides, *Pf*DGAT, and lipid body peaked at the schizont stage within *P. falciparum*, followed by FA and triglyceride release [81], showing *Pf*DGAT is also proposed as a potential target. Nevertheless, triglycerides synthesis are not necessary for the survival of *Leishmania major* [82].

Both the enzymes involved in lipid synthesis de novo and those involved in host scavenging are crucial for *T. gondii*. For instance, loss of *Tg*PDH in the FAS II pathway or *Tg*PTS in phospholipid metabolism can be compensated by other pathways [18, 32]. Lack of PE and PS would induce *T. gondii* to take up these lipid components by P4-ATPase [35], suggesting that external lipid uptake can be used as a compensation mechanism for internal lipid deficiencies. In turn, the IPC synthase activity in sphingomyelin de novo synthesis pathway makes extrinsic lipid ingestions dispensable [49]. However, cholesterol in *T. gondii* can only be taken up by the host [56], and no literature has yet reported its compensatory mechanism, revealing its unique status. Overall, identification and functional analysis of lipid-translocating proteins on PPM or PVM, as well as enzymes involved in transport of lipid components within *T. gondii*, will be vital for the recognition of the molecular mechanisms of parasite–host interactions [72, 83]. These characteristics enable the development of targeted drugs that can effectively induce the death of *T. gondii*, thereby offering an intriguing therapy for toxoplasmosis [9].

## Data Availability

Not applicable.

## Abbreviations

*T*.* gondii*: *Toxoplasma gondii*
FAs: Fatty acids
FAS II: Type II fatty acid synthesis
ER: Endoplasmic reticulum
FAE: Fatty acid elongation
FAS I: Type I fatty acid synthesis
FAS: Fatty acid synthase
LCFA: Long-chain fatty acids
PKSs: Polyketide synthases
C16:0: Palmitic acid
C14:0: Myristic acid
ACP: Acyl carrier protein
FabD: Malonyl-CoA: ACP transcylase
FabH: β-Ketoacyl-ACP synthase III
FabZ: β-Hydroxyacyl-ACP dehydratase
FabG: 3-Oxoacyl-ACP reductase
FabI: Enoyl-ACP reductase
PDH: Apicoplast pyruvate dehydrogenase
Act-CoA: Acetyl-CoA
ELOs: Fatty acid elongases
DEH: Hydroxyacyl-CoA dehydratase
ECR: Enoyl-CoA reductase
PI: Phosphatidyl inositol
PE: Phosphatidyl ethanolamine
PEP: Phosphoenolpyruvate
APT: Apicoplast phosphate transporter
PYK2: Pyruvate kinase 2
Pyr: Pyruvate
Mal-CoA: Malonyl-CoA
ACCase I: Acetyl-CoA carboxylase I
Glu: Glucose
GT1: Glucose transporter 1
ELO-A: Fatty acid elongase A
ELO-B: Fatty acid elongase B
ELO-C: Fatty acid elongase C
KCR: Ketoacyl CoA reductases
ACCase II: Acetyl-CoA carboxylase II
TCA: Citric acid cycle
LCACoAs: Long-chain acyl-CoA
ACBP1: Acyl-CoA transporters
PC: Phosphatidyl choline
PS: Phosphatidyl serine
PA: Phosphatidic acid
DG: Diacylglycerol
DGKs: Diacylglycerol kinases
CDP: Cytidinediphosphate
CK: Choline kinase
PT: Phosphatidyl threonine
PG: Phosphatidyl glycerol
SPT: Serine palmitoyl transferase
PLP: Pyridoxal phosphate
IPC: Inositol phosphorylceramide
AUR1p: Yeast IPC synthase
CTP: Cytosine triphosphate
CDP-DG: Cytosine diphosphate diacylglycerol
PAP: Phosphatidic acid phosphatase
CDS: Cytosine diphosphate diacylglycerol synthetase
PCS: Phosphatidyl choline synthetase
EPT: Phosphatidyl ethanolamine synthetase
PSS: Phosphatidyl serine synthetase
PSD: Phosphatidyl serine decarboxylase
PTS: Phosphatidyl threonine synthetase
PIS: Phosphatidyl inositol synthetase
PGPS: Phosphatidylglycerol phosphate synthase
PGPP: Phosphatidylglycerol phosphate phosphatase
P4-ATPase: P4 plasma membrane flipping ATPase
PPM: Parasite plasma membrane
PVM: Parasitophorous vacuole membrane
LDL: Low-density lipoprotein
PV: Parasitophorous vacuole
HOST: Host organelle-sequestering tubulostructures
NPC: Niemann-Pick type C
IVN: Intravacuolar network
LCAT: Lecithin: cholesterol acyltransferase
PLA2: Phospholipase A2
P-gp: P-glycoprotein
ABC: ATP-binding cassette
ACAT: Acyl-CoA: cholesterol acyltransferase
DGAT: Diacylgycerolacyl transferase
GRA: Dense granule protein
ROP: Rhoptry protein
ABCG: ATP-binding cassette G subfamily
LPA: Lysophosphatidic acid
G3P: Glycerol 3-phosphate
C: Cholesterol
CE: Cholesteryl esters
ATS1: Glycerol 3-phosphate acyltransferase
ASH: Active serine hydrolases
GPAT: Glycerol-3-phospahte-acyl transferase
CDPK: Calcium dependent protein kinase
Etn(Me)(2): Dimethylethanolamine
*P. falciparum*: *Plasmodium falciparum*
*T. brucei*: *Trypanosoma brucei*
*T*. *cruzi*: *Trypanosoma* *cruzi*
*L*. *donovani*: *Leishmania* *donovani*
*E*. *falciformis*: *Eimeria* *falciformis*
*N. caninum*: *Neospora* *caninum*
